# The Effect of Neutrophil-Derived Products on the Function of Leukocytes Obtained after Titanium Implantation in the Ovine Model

**DOI:** 10.3390/ani11123569

**Published:** 2021-12-15

**Authors:** Joanna Zdziennicka, Joanna Wessely-Szponder, Grzegorz Starobrat, Andrzej Junkuszew

**Affiliations:** 1 Sub-Department of Pathophysiology, Department of Preclinical Veterinary Sciences, Faculty of Veterinary Medicine, University of Life Sciences, 20-033 Lublin, Poland; joanna.michalska15@gmail.com; 2 Children Orthopaedic Department, Faculty of Medicine, Medical University of Lublin, 20-093 Lublin, Poland; grzegorz.starobrat@umlub.pl; 3 Institute of Animal Breeding and Biodiversity Conservation, Faculty of Animal Sciences and Bioeconomy, University of Life Sciences, 20-950 Lublin, Poland; andrzej.junkuszew@up.lublin.pl

**Keywords:** sheep, antimicrobial peptides, titanium implant, monocyte-derived macrophages, neutrophil degranulation products

## Abstract

**Simple Summary:**

Titanium is one of the most commonly used biomaterials for implantation as a part of the orthopedic procedures. However, this biomaterial can cause an excessive inflammatory response, even leading to rejection of the implant. Therefore, the aim of our study was to assess the overall organism response after insertion of Ti implant and the activity of neutrophils and monocyte-derived macrophages (MDM), to evaluate the possible negative effect of this biomaterial on the host cells. Our study revealed that insertion of the Ti implant did not evoke systemic inflammatory response or activation of leukocytes. Additionally, we evaluated the activity of neutrophils and MDM after stimulation with autologous neutrophil products, namely, antimicrobial neutrophil extract and neutrophil degranulation product as two potential regulators of inflammatory response. Antimicrobial neutrophil extract appeared to be a factor causing the decrease of secretory neutrophil response and polarization of MDM towards pro-resolving phenotype, whereas the neutrophil degranulation product acted as pro-inflammatory.

**Abstract:**

Titanium (Ti) is currently the most common biomaterial used for orthopedic implants; however, these implants may cause deleterious immune response. To investigate the possible mechanisms involved in excessive inflammation, we assessed the activity of neutrophils and monocyte-derived macrophages (MDMs) during the insertion of the Ti implant in a sheep model. The study was conducted on 12 sheep, 4 of which were control animals and 8 were in the experimental group with inserted Ti implant. Neutrophil secretory response was estimated at two time points T0 before surgery and T1 1 h after implantation and was based on the release of enzymes from neutrophil granules and reactive oxygen and nitrogen species (RONS) generation. MDM function was evaluated 5 months after implantation, on the basis of RONS generation arginase activity and morphological changes. Moreover, the influence of some autologous neutrophil derived products, namely, antimicrobial neutrophil extract (ANE) and neutrophil degranulation products (DGP) on leukocytes was estimated. Our study revealed that Ti implant insertion did not cause any adverse effects up to 5 months after surgical procedure. Stimulation of neutrophil cultures with ANE decreased the enzyme release as well as superoxide generation. Treatment of MDM with ANE diminished superoxide and NO generation and increased arginase activity. On the other hand, MDM stimulated with DGP showed elevated superoxide and NO generation as well as decreased arginase activity. To summarize, ANE exerted an anti-inflammatory and pro-resolving effect on studied leukocytes, whereas DGP acted as pro-inflammatory.

## 1. Introduction

Using various metal implants is necessary to improve healing and restore tissue integrity in many orthopedic disorders. Implants may, however, spark an excessive immune response, leading to high failure rates accompanied with immune implant rejection [1]. Despite completed bioavailability tests, biomaterials used for implantation may be responsible for the induction of adverse immune reactions, resulting in excessive inflammation and impairment of healing that leads to tissue damage or even isolation and rejection of the implant. A better understanding of the interactions between the biomaterial and the organism is strongly needed to develop solutions to overcome the adverse effects after using of biomaterials [2].

The role of neutrophils in the bone healing process remains controversial; some authors postulated a negative influence of neutrophils on bone regeneration because their depletion improved fracture healing. They proposed that neutrophils could cause tissue damage by release of collagenase, elastase, free radicals and arachidonic acid. Indeed, this activity might be beneficial for intramembranous bone formation, but diaphyseal fracture healing might be delayed, because cartilaginous callus formation is essential for this process. More recent studies indicated that a balanced neutrophil activation is important for fracture healing [3]. Therefore, it is vitally important to evaluate the role of neutrophils as the first responders to implanted biomaterials [4].

Neutrophils and blood monocytes invade the injury site during the course of tissue repair, where they participate in host defense, phagocytosis of necrotic tissue, and secretion of paracrine factors. Macrophages, in turn, are essential for the regeneration, repair, and remodeling in many tissues. However, chronic inflammation and persistent macrophages accumulation are often associated with tissue destruction and fibrosis [5].

Metal materials are commonly used in orthopedic and dental implants; however, recent evidences suggest that these biomaterials induce an immunomodulatory interaction with the host. Therefore, it was assumed that materials such as titanium (Ti) are not inert upon contact with host bone [2,6]. Ti is considered to be biocompatible because it has a low electrical conductivity which contributes to the electrochemical oxidation leading to the formation of a thin passive oxide layer [7]. However, the surface of implant significantly influences the Ti content found in the bone after implantation. The surface structure of the implant is important for the amount of Ti released, while total area and diameter of the implant are of less importance [8,9]. Thus, using metal implants evokes problems with biocompatibility in the context of bone–metal interface processes and ion release [6]. The outcome of biomaterial implantation depends on the extent of the foreign body reaction (FBR) and related immune and inflammatory responses [10]. All implanted materials are able to induce FBR, which is primarily mediated by macrophages. The reaction severity may depend not only on the nature of the implanted material, its structure and surface topography, but also on the implant organism’s individual reactions [11]. Some authors previously showed that titanium ions form particles that induced monocyte infiltration, and, through the activation of macrophages, can act as secondary pro-inflammatory cytokines to release stimuli [11,12,13,14]. For these reasons, current studies are focusing on understanding these interactions in order to improve the properties of implanted biomaterials. However, the long-term immune recognition of implants as well as other precise mechanisms of this response have not been fully understood yet [6].

Measuring the response of some immune cells to foreign biomaterials, including cell viability and activation studies at different time points, is the most common method for characterizing in vitro immunogenic response, with a pivotal role for clinical outcomes and potential therapeutic application [1].

Titanium is a generally accepted biomaterial as a relatively inert substance with minimal side effects, and it is used in many medical fields among other in orthopedics implants. However, with the increased application of Ti, the concerns over the safety of this biomaterial also increases. Some recent studies suggest that, under some circumstances, the presence of Ti particles may be harmful, especially following frictional wear of medical prostheses or of screws used in plate fixation during orthopedic procedures. The particles and ions released from Ti implant can be deposited in surrounding tissues causing inflammatory reactions, hypersensitivity, and/or can lead to toxic reactions [15]. Thus, there is a need for interest about Ti safety and potential dangers [15,16]. For these reasons we decided to investigate the role of two neutrophil-derived products as potential modifiers of the white blood cells (WBC) system response during implantation of biomaterial. The results from the study conducted by Frisken et al. indicate that sheep is an excellent model for following the biological changes associated with successful or failed insertion of Ti implants [16]. Sheep are also widely accepted as a large animal model for translating potential musculoskeletal injury and repair therapies [17,18]. Therefore, we decided to use this model for Ti implant insertion into the tibia and to evaluate two ovine neutrophil products: antimicrobial neutrophil extract (ANE) and neutrophil degranulation products (DGP) for potential enhancement of healing. Ovine neutrophil extract is composed of some cathelicidins, namely, SMAP-29, SMAP34, OaBac5, OaBac6, 5 Oabac7.5, and OaBac11 [19]. It has been previously considered as an alternative for antibiotics [20,21], whereas bovine DGP was previously evaluated by Hussen et al. [21] as a potential stimulator of monocytes and monocyte–macrophage differentiation. To broaden the knowledge about biomaterial–host interactions on the basis of WBC response, we evaluated the immediate and long-term leukocyte response to Ti implant insertion in this animal model. In light of the fact that excessive (WBC) response may cause disturbances in the healing processes, up to implant rejection, our first aim was to assess neutrophil and monocyte-derived macrophages (MDM) activity during insertion of Ti implants into the proximal tibial physis in a sheep model. In this study, the overall responses involved in surgical procedures, implant insertion, and postoperative treatment were taken into account. For the assessment of systemic immune response, plasma concentration of acute phase proteins (APP) was measured at appropriate time points. Neutrophil activity was evaluated during the inflammatory phase of the healing process, whereas MDM assessment was conducted during the long-term immune recognition of implants. Our second aim was to evaluate two neutrophil-derived autologous products i.e., ANE and DGP, as potential modifiers of leukocyte response during Ti implant insertion. The obtained results have the potential for application in the regulation of inflammatory response during the repair process and to decrease excessive inflammation.

## 2. Materials and Methods

### 2.1. Animals and Study Design

The study was conducted in a group of 12 sheep, females, BCP local breed, 4 months old, approximately 20 kg body weight, from the Bezek Experimental Farm, University of Life Sciences in Lublin. Animal management and surgical protocol were approved by the local Ethics Committee number II in Lublin (No 84/2019). The animals were randomly divided into two groups, a control group (*n* = 4) and an experimental group (*n* = 8). All sheep from the experimental group had a Ti implant inserted into the tibia; sheep from the control group were used for comparison on long-term effect of Ti implant on MDM population.

### 2.2. Surgical Procedure

**Figure 1:**
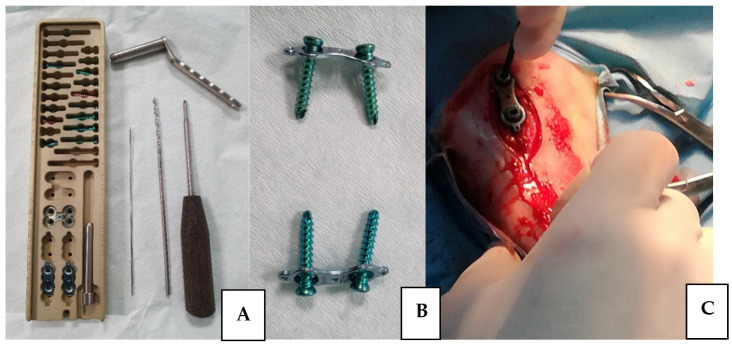
(**A**) Photography of the standard eight plate systems equipped with elastic plate. (**B**) Two screws and the implant which were used in the experiment. (**C**) The implantation of the eight plate system in the ovine tibia.

After premedication with intramuscular injection of xylazine (0.1 mg/kg) and Butorphanol (0.1 mg/kg), the surgical area was aseptically cleaned, and the standard surgical approach for proximal tibia was performed. Then, the commercial sterile eight plate system for pediatric usage with elastic plate and two screws was implanted [22] (Figure 1).

Postoperatively, all sheep received antibiotic (Combi-ject 200,000 IU/mL Penicillin and 200 mg/mL Streptomycin) intramuscularly as well as Melovem (Meloxicam 5%, Dopharma Research B.V. 1.2 mL SC) as analgesic. For two weeks after surgery, a full clinical examination with routine post-operative inspection of surgical wounds and vital parameters recording was conducted every day. Thereafter, these procedures were conducted once a week up to 5 months after surgery.

### 2.3. Blood Sampling, Cell Cultures, and Blood-Derived Products

Blood for hematological assays was collected from the jugular vein seven days before the experimental procedure. Blood was collected into tubes containing EDTA as an anticoagulant. A complete blood count was performed using an Abacus Junior Vet analyzer (Diatron, Budapest, Hungary) and hematological parameters in all animals were within the reference ranges. At the same time, blood aliquots of approximately 25 mL with 3.8% sodium citrate as anticoagulant were obtained for preparing both blood-derived products, namely, ANE and DGP.

ANE was prepared as previously described [23]. Blood was centrifuged once red blood cells underwent lysis with 0.83% ammonium chloride. Neutrophils (>75% purity in May–Grunwald–Giemsa-stained preparations) were resuspended in modified phosphate saline buffer (PBS, Biomed Lublin, Lublin, Poland and then homogenized with a DIAX 900 (Heidolph, Schwabach, Germany) to release the neutrophil granules. These granules were collected, suspended in 10% acetic acid, and stirred overnight to extract antimicrobial peptides. After determination of peptide concentration [24], the portions of ANE containing 40 μg/mL of peptides were prepared, lyophilized, and stored at −70 °C for further use.

The obtained ANE was used for stimulation of neutrophil or MDM cultures to assess their activity. To achieve this, immediately before the experiments appropriate amounts of lyophilized samples were resuspended in a sterile medium to final desired concentrations and added to cultures. ANE concentrations of 0, 4, 40, and 100 μg/mL were used to assess potential toxicity against ovine cells; for assessment of leukocytes in vitro activity, the concentrations of 0 and 40 μg/mL were used.

DGP preparation relied on the degranulation of isolated neutrophils, induced by *N* formylmethionyl-leucyl-phenylalanine (fMLP, 1μg/mL, Sigma-Aldrich, Poznan, Poland). After 10 min of incubation at 37 °C, neutrophils were centrifuged (300× *g*, 10 min) and cell free supernatant containing DGP was collected and stored at −20 °C until later use [21].

### 2.4. Evaluation of Cytotoxicity and Hemolytic Properties of ANE

A 20 mL blood sample from each sheep was used for preparing approximately 4 mg of neutrophil extract (ANE), which was lyophilized. A part of this lyophilizate was used to test for potential cytotoxic and hemolytic activity against host cells.

The effect of ANE on neutrophil viability was determined using an MTT test (3-[4,5-dimethyl-2-thiazolyl]-2,5-diphenyl-2H-tetrazolium bromide-MTT), which is based on the conversion of MTT tetrazolium salt by mitochondrial dehydrogenase to a formazan color product as measured at an absorbance 169 of 565 nm [25]. Following stimulation with different ANE concentrations (0, 4, 40, and 100 μg/mL), neutrophils were cultured at 37 °C with 5% CO_2_ for 24 h, then the cells were incubated with 5 mg/mL MTT in PBS for 3 h, lysed in 10% SDS overnight, and optical density was measured. The obtained values were calculated as a percentage of 100% activity of cells without stimulation. Cell viability was assessed in triplicate.

To assay hemolytic activity, freshly drawn ovine blood was collected into an EDTA tube and plasma was removed by centrifugation (500× *g*, 10 min). Erythrocytes were rinsed three times with PBS and resuspended at a concentration of 2%. Then, 200 μL of erythrocyte suspensions with various ANE concentrations (4, 40 and 100 μg/mL) were incubated at 37 °C for 30 min in 96-well microtiter plates. Triton X-100 (1%, Sigma-Aldrich Poznan, Poland) was used as the positive control for 100% hemolysis and PBS was used as the negative control. Absorption of supernatants was measured at 405 nm, using a BioTek EL800 microplate reader (BioTek, Janki, Poland). All assays were performed in triplicate [24,26,27].

### 2.5. In Vitro Neutrophil Activity

**Figure 2:**
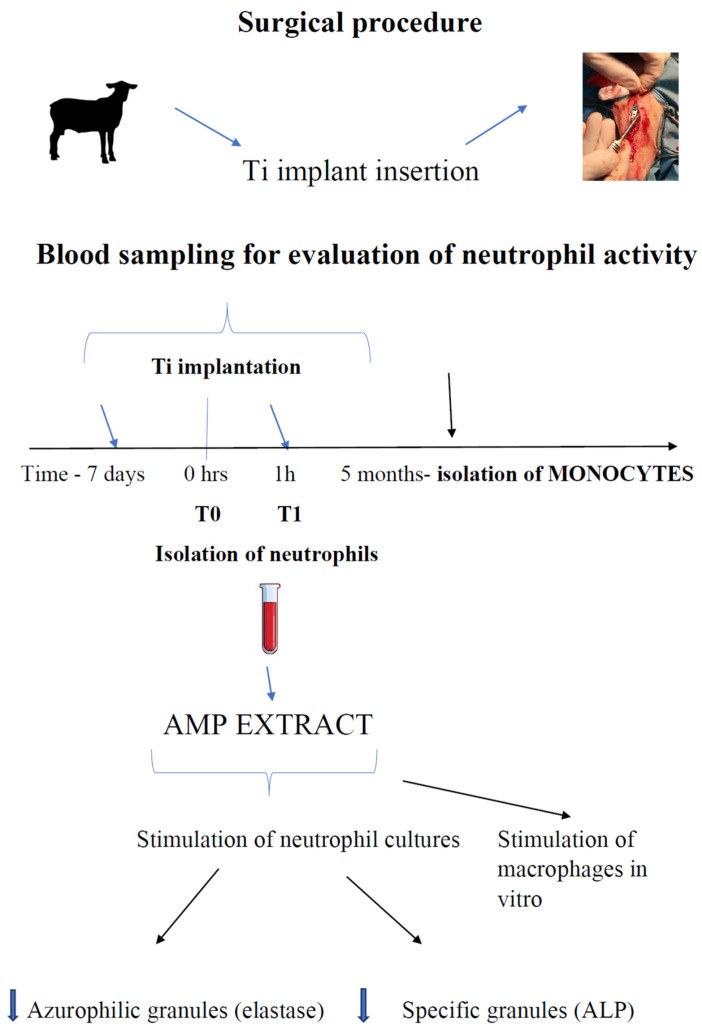
Preparation of ANE (AMP extract) and timeline of blood sampling for isolation of leukocytes from circulating blood.

Blood samples for neutrophil in vitro assays were collected into EDTA tubes at two time points: first, T0—7 days before surgery; second, T1—1 h after surgery (Figure 2).

The cell suspensions prepared at a density of 2.0 × 10^6^ cells/mL were supplemented with ANE at a concentration of 40 μg/mL and marked as stimulated or the control group with PBS (marked as unstimulated) and both groups were incubated for 30 min and for 22 h at 37 °C in the presence of 5% CO_2_.

Enzyme release from azurophilic granules was estimated on the basis of elastase activity and was measured spectrometrically on the basis of cleavage of azocasein (Sigma-Aldrich) using a microplate reader BioTek EL800 (BioTek, Janki, Poland). Alkaline phosphatase (ALP) release, as a marker of specific granule response, was determined after 10 min incubation at 25 °C with substrate 4-nitrophenyl phosphate disodium salt hexahydrate (Sigma-Aldrich, Poznan, Poland). Nitric oxide (NO) level was determined by means of the Griess reaction, and superoxide anion generation was measured as described previously [28].

### 2.6. Monocyte Isolation, Generation of Monocyte-Derived Macrophages, Functional and Morphological Analysis

Approximately 10 mL of blood was taken from each sheep 5 months after implantation of biomaterial to obtain MDM; additionally, the blood from healthy sheep (*n* = 4) was sampled to obtain a control group for comparison. Mononuclear cells fraction (MNC) was isolated by gradient centrifugation over Histopaque-1077 (Sigma-Aldrich, Poznan, Poland). To obtain macrophages, MNC were immediately seeded at a concentration of 1.0 × 10^6^ cells/mL into 96-well flat-bottomed tissue culture plates (Nunc, Termofisher Scientific, Warsaw, Poland) and cultured at 37 °C and 5% CO_2_ for 72 h in Dulbecco’s Modified Eagle’s Medium (DMEM) with 10% bovine calf serum (BCS, Biomed, Lublin, Poland) [28]. The cultures described as BCS were left without additional stimulation. Other cultures were stimulated as follows: the ANE group was stimulated with 40 μg/mL of ANE, others were stimulated with DGP (DGP group), or with 1 μg/mL lipopolysaccharide from *Escherichia coli* serotype 055:B5 (Sigma-Aldrich, Poznan, Poland), as an LPS group, or with dexamethasone (DEX group) at a concentration of 100 nM. All these cultures were incubated for 3 days at 37 °C and 5% CO_2_. During this period, cultures underwent daily functional analysis on the basis of NO generation and superoxide generation. Arginase activity was assessed at the end of the experiment (after 72 h of incubation).

Superoxide production was measured using the method described previously [29]. The NO level was determined using the Griess reaction as described [29]. Arginase activity was assessed on the basis of the concentration of urea which was measured after addition of 40 μL of α-isonitrosopropiophenone (Sigma-Aldrich, Poznan, Poland), followed by heating at 100 °C for 30 min [23]. Microscopic analysis of the morphology was conducted using a reversed phase microscope (Olympus, Warsaw, Poland).

### 2.7. Assessment of Acute Phase Proteins (APP)

Fibrinogen and haptoglobin plasma concentrations were evaluated as described previously [30] at three time points: A—before, B—1h after, and C—7 days after Ti implant insertion into the tibia.

### 2.8. Statistical Analysis

Data from neutrophil and MDM activity, as well as assessment of APP response were analyzed using Statistica 13.1 (StatSoft, Krakow, Poland). All results represented the mean and standard error (SE) of their respective groups. Significance was calculated using a one-way ANOVA followed by a Tukey’s test. Differences were considered significant at *p* < 0.05.

## 3. Results

### 3.1. Clinical Findings

No adverse effects were observed in the course of the experiment up to 5 months after implantation of Ti implant into ovine tibia.

### 3.2. Evaluation of ANE, Hemolysis, and Cellular Toxicity

**Figure 3:**
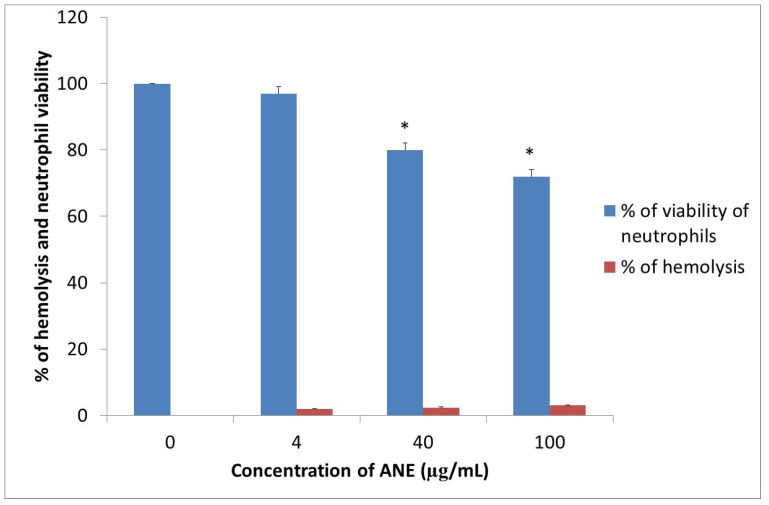
Test of potential cytotoxic and hemolytic ANE activity against host cells (neutrophils and erythrocytes, respectively). ANE was used at concentrations of 4, 40, and 100 µg/mL. Dose-dependent reduction in viability and increased hemolysis were observed (* *p* < 0.05 compared to 0 µg/mL).

Our study revealed low cellular toxicity of ANE measured as a decrease in the viability of ovine neutrophils. At the highest concentration, namely, 100 μg/mL, cell viability was reduced to 72%. During hemolytic activity studies, we estimated that ovine erythrocytes were resistant to ANE at concentrations of 4 and 40 μg/mL, the concentration of 100 μg/mL only caused a slight lysis, reaching up to 3% (Figure 3).

### 3.3. Response of Neutrophils Obtained from Sheep before and after Implantation, Unstimulated or Stimulated with ANE

**Figure 4:**
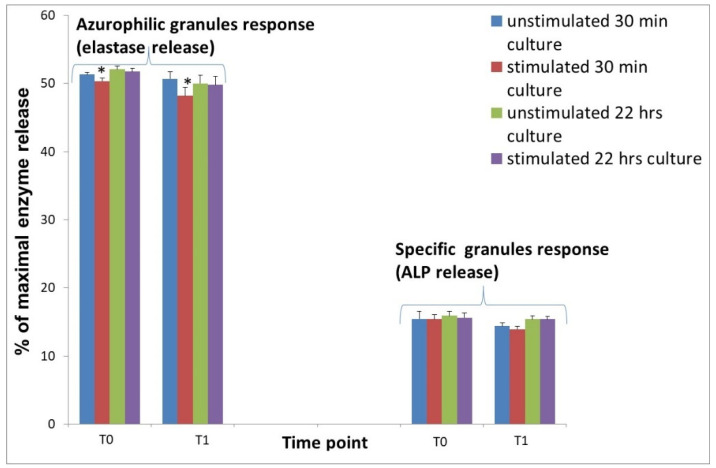
Release of elastase and alkaline phosphatase (ALP) from neutrophils isolated from sheep 7 days before implantation (T0) and 1 h (T1) after Ti implant insertion into the tibia. Cultures of isolated neutrophils were left without stimulation (unstimulated) or stimulated with 40 µg ANE for 30 min and for 22 h. Data represents the mean ± SE of 8 sheep with duplicate measurements. There are significant differences between mean values marked with * (*p* < 0.05).

The differences in activity of neutrophils isolated before and after implantation of biomaterial were insignificant. On the other hand, changes were noted after the treatment of the cultures with ANE. In neutrophil cultures obtained after implantation of biomaterial (T1), the stimulation with ANE for 30 min caused significant (*p* < 0.05) decrease of elastase release from 50.7 ± 0.8 to 48.2 ± 0.48% of maximal enzyme release. In cultures after 22 h incubation, changes were insignificant (Figure 4). ALP release decreased at T0 and T1 after stimulation with ANE for 30 min, but changes were insignificant (Figure 4).

**Figure 5:**
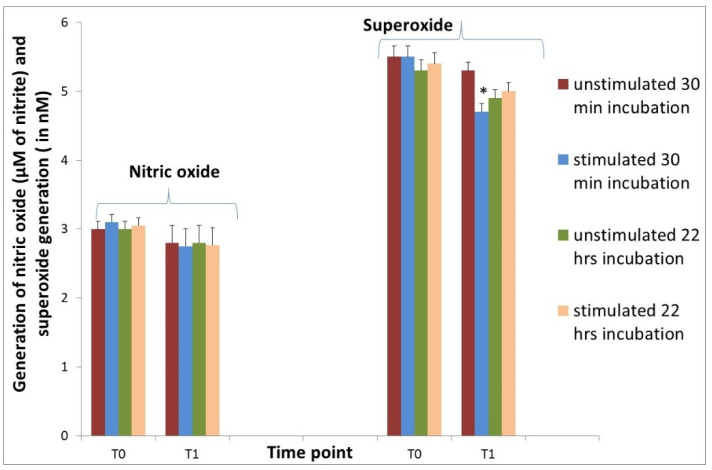
Generation of superoxide and nitric oxide from neutrophils isolated from sheep 7 days before (T0) and 1 h (T1) after Ti implant insertion into the tibia. Cultures without stimulation (unstimulated) and after stimulation with ANE (stimulated) for 30 min and for 22 h. Data represent the mean ± SE of 8 sheep in duplicate measurements. There are significant differences between mean values marked with * (*p* < 0.05).

Superoxide generation under the influence of ANE was decreased at T1 after 30 min in comparison with values without stimulation. Changes in NO generation were insignificant (Figure 5).

### 3.4. MDMs Activity and Morphology In Vitro

Initially, we compared MDM obtained from healthy sheep (*n* = 4, control group) with MDM from a group of sheep 5 months after implantation (*n* = 8, experimental group). We did not observe any significant differences between MDM isolated from the control and experimental sheep. Then, we evaluated differences between both groups after treatment with different stimulations. Part of the mature macrophages was described as BCS and were left without any additional stimulation for comparison with other cultures treated with different stimulators. One part of MDM was stimulated with 40 μg/mL of ANE to obtain the ANE group, another part was stimulated with DGP (DGP group), or with 1 μg/mL LPS (LPS group), or with dexamethasone (DEX group) at a concentration of 100 nM. We found no significant differences between MDM from the control and the experimental sheep. However, there were noticeable differences in MDM activity and morphological features that only resulted from the type of stimulus used.

### 3.5. MDMs Response to ANE

**Figure 6:**
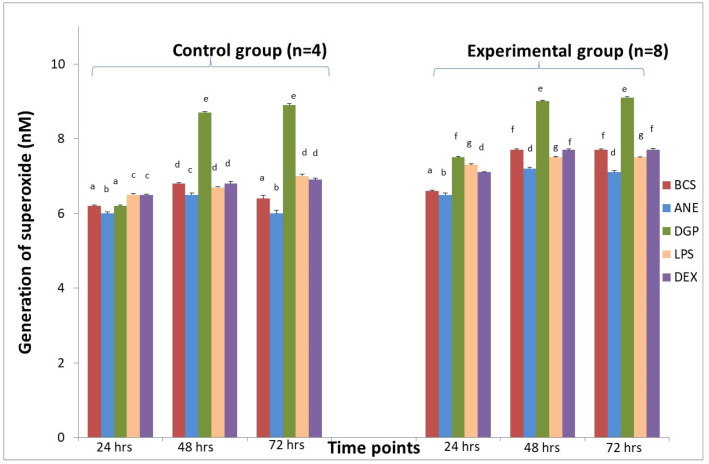
The effect of different stimulators on superoxide generation by macrophages derived from sheep in the control group (*n* = 4) and in the experimental group (*n* = 8) during insertion of Ti implant into the tibia. BCS—macrophages stimulated only with bovine calf serum, ANE—antimicrobial neutrophil extract, DGP—neutrophil degranulation product, LPS—lipopolysaccharide, DEX—dexamethasone. Values are mean ± SE obtained from each separate experiment conducted in both groups of sheep. There are significant differences between mean values marked with different letters (*p* < 0.05) compared to BCS group in each time point.

Superoxide generation by macrophages treated with ANE was significantly (*p* < 0.05) lower after 48 and 72 h incubation in sheep from both the control and experimental group, in comparison with MDMs without additional stimulation (BCS group) (Figure 6).

**Figure 7:**
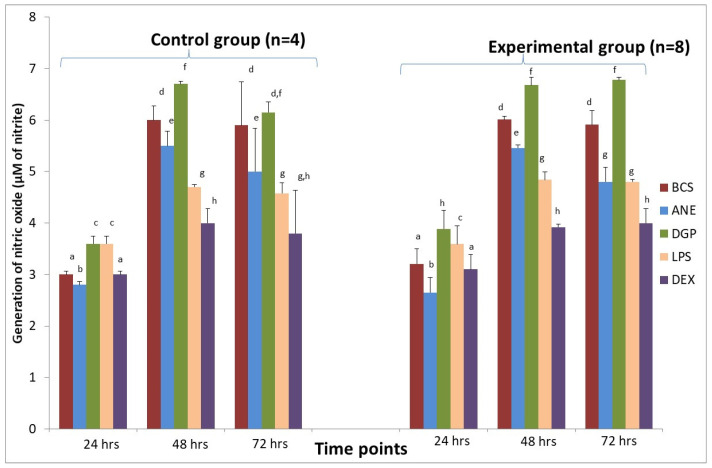
The effect of different stimulators on nitric oxide (NO) generation by macrophages derived from sheep in the control group (*n* = 4) and in the experimental group (*n* = 8) during insertion of Ti implant into the tibia. BCS—macrophages stimulated only with bovine calf serum, ANE—antimicrobial neutrophil extract, DGP—neutrophil degranulation product, LPS—lipopolysaccharide, DEX—dexamethasone. Values are mean ± SE obtained from each separate experiment conducted in both groups of sheep. There are significant differences between mean values marked with different letters (*p* < 0.05). compared to BCS group in each time point.

We also observed a decrease in the generation of NO in MDM stimulated with ANE in both groups obtained from the control and the experimental sheep (Figure 7).

**Figure 8:**
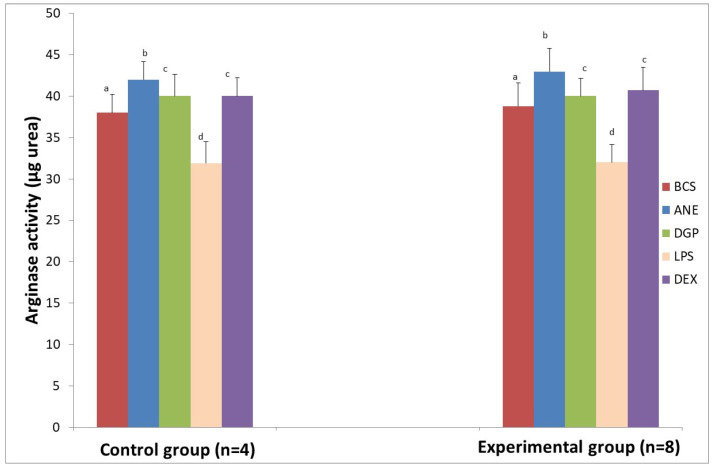
Arginase activity in lysates of MDMs isolated from sheep during implantation of Ti implant into the tibia in the control group (*n* = 4) and the experimental group (*n* = 8). BCS—macrophages stimulated only with bovine calf serum, ANE—antimicrobial neutrophil extract, DGP—neutrophil degranulation product, LPS—lipopolysaccharide, DEX—dexamethasone. Values are mean ± SE obtained from each separate experiment conducted in both groups of sheep. There are significant differences between mean values marked with different letters (*p* < 0.05) compared to BCS group.

Together with a decrease of reactive oxygen and nitrogen species (RONS), an elevated arginase activity was noted after MDM stimulation with ANE. We found that arginase activity increased from 38.00 ± 2.2 μg urea to 42.00 ± 3.00 μg urea (*p* < 0.05) in the sheep from the control group, whereas in the experimental group it increased from 38.81 ± 2.63 μg urea to 42.98 ± 3.85 μg urea (*p* < 0.05) (Figure 8).

**Figure 9:**
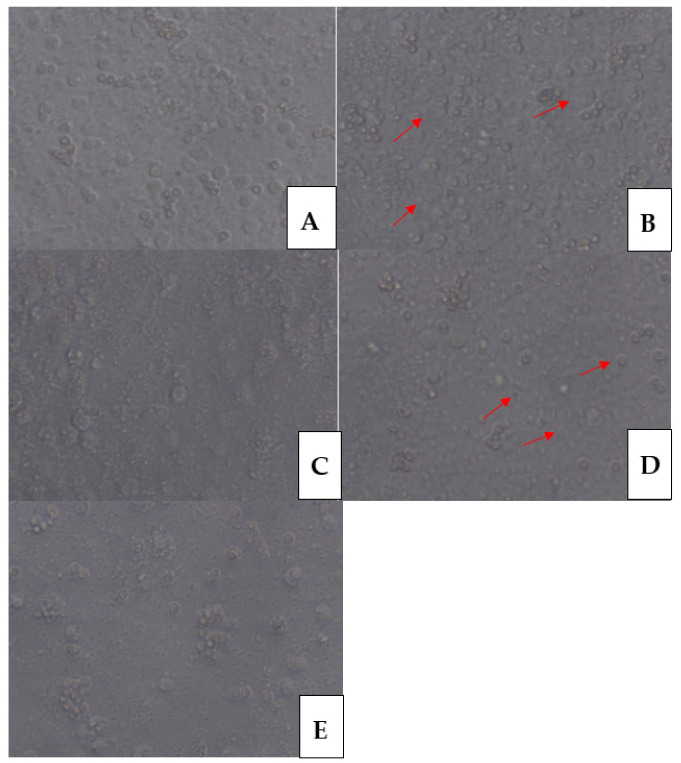
Representative phase contrast microscopical images revealing the effects of different stimulators on the morphology of the resulting macrophages after 3 days of culture. (**A**) MDMs left without any additional stimulation were described as BCS. (**B**) MDMs stimulated with ANE (40 μg/mL). Black arrows indicate ‘fried egg’-shaped phenotype. (**C**) MDMs stimulated with LPS (1 μg/mL). (**D**) MDMs stimulated with DGP. Red arrows indicate filopodia. (**E**) MDMs stimulated with DEX (100 nM). Original magnification ×40.

Morphological changes were suggestive of an anti-inflammatory pro-resolving phenotype (Figure 9).

### 3.6. MDMs Response to DGP

After stimulation with DGP, the pro-inflammatory response of MDM was estimated on the basis of increased superoxide generation (Figure 6) and NO generation measured in both groups after 48 and 72 h incubation (Figure 7). The highest superoxide generation after stimulation with DGP was noted in the last measurement when the value achieved was 8.9 ± 0.02 nM in MDM from the control animals and 9.1 ± 0.03 nM in MDM from the experimental animals (Figure 6). Arginase activity after stimulation with DGP was lower in comparison with MDM stimulated with ANE, and was similar to cultures treated with DEX, whereas LPS addition caused a significant decrease of arginase activity (*p* < 0.01) (Figure 8). The morphology of MDM stimulated with DGP was pro-inflammatory with visible filopodia (Figure 9).

### 3.7. Acute Phase Proteins (APP)

Immediately after surgery (B), plasma concentration of both evaluated APP—fibrinogen and haptoglobin—increased compared to the values before surgery (A), and 7 days after insertion of the Ti implant (C) the level of both APP decreased to values similar to range before procedure (Table 1).

## 4. Discussion

In this study, we used an ovine biomaterial implantation model, and the primary aim of the experiment was to evaluate response of two subpopulations of the white blood cells system: neutrophils and MDM, to the implantation of a Ti plate into the tibia. We evaluated elastase and ALP release as markers of degranulation of azurophilic and specific granules, respectively. Additionally, RONS generation was a marker of neutrophil activity. The enzymatic response remained unchanged after implantation; thus, circulatory activation of neutrophils did not appear.

Previously, neutrophil response to implantation was assessed in rabbit neutrophils during osteochondral grafting, when increased activity of these cells was noted two hours and three days after surgery and was related to increased acute phase response [30]. Some authors estimated that systemic inflammatory response may be induced by trauma [4,31,32]. Moreover, neutrophils are known to react with Ti ions that stimulate neutrophils to produce superoxide anions. Ti nanoparticles could be pro-inflammatory and cytotoxic at higher concentrations [13]. Innate immune system responses to biomaterial wear particles induce osteolysis as a significant adverse effect, which leads to loosening of the implant components and implantation failure [33]. No excessive systemic neutrophil activation was identified in our experiment after implantation, and we did not observe any adverse reactions to Ti implants. Up to five months after implantation, we did not find any clinical signs that would be indicative of implant rejection or implantation failure and any systemic inflammatory response on the basis of APP. In our previous experiment on ovine model of insertion of a Ti plate, we evaluated the neutrophil secretory response in other time points, namely, 2 h, 24 h, 4 months, and 10 months after implantation, with similar results [22].

Early response of MDM on Ti implant insertion was evaluated previously [34]. In the course of this experiment, we did not observe increased activity of MDM obtained from sheep after implantation when compared to control animals. We decided to evaluate MDM response 5 months after implantation to reveal any possible effects of the implant particles on host response. In our previous experiment, MDM response was evaluated during Ti implantation into a rabbit tibia [23] and the MDM activity was similar in rabbits before and after implantation. However, a potential influence of Ti ions on MDM had to be taken into account, because previously certain authors reported Ti toxicity [15], allergic reactions [33], as well as activation of MDM after contact with Ti implant [8,11,12,35], and the influence of the implant surface on release and bone deposition of Ti particles was underlined [35].

The second part of the experiment included preparation of the autologous neutrophil derived product ANE (a mixture of ovine antimicrobial peptides), which was then used for in vitro stimulation of neutrophils and MDM. Numerous antimicrobial peptides (AMP) from a large variety of animal sources have been isolated and evaluated to date. It turned out that these peptides play an important role in host defense. Ovine extract has previously been considered a therapeutic agent against some ovine pathogens and its possible use in humans against cystic fibrosis or oral infections has been considered [19]. However, the potential cytotoxicity of AMP limited its widespread clinical use. For example, the clinical application of porcine cathelicidin (PG-1), which had promising antimicrobial properties, has been restricted because of its high mammalian toxicity, causing approximately 37% hemolysis at concentrations of 100 μg/mL [36]. For this reason, potential ANE toxicity was evaluated before starting the stimulation experiments.

Results clearly indicate that ANE demonstrated low hemolytic activity at the doses used, with the highest hemolysis reaching 3% at 100 μg/mL. Hemolytic effects of ANE on ovine erythrocytes have not been previously evaluated. According to Skerlevaj et al. [24], ovine erythrocytes are resistant to ovine cathelicidin SMAP-29, which caused only 3% lysis at 80 μg/mL concentrations. Moreover, our study revealed that ANE at the concentration used caused only a slight decrease of neutrophil viability from 100% at a negative control to 72% at a concentration of 100 μg/mL. The toxicity of the mixture of ovine cathelicidins present in ANE to mammalian cells has not been previously evaluated; however, some authors studied the influence of other AMP against different cells and obtained varied results. According to Catrina et al. [37], some antibacterial peptides are not toxic for resting T cells. On the other hand, Arabiou et al. [25], estimated that human defensin NHP1 at concentrations from 50 μg/mL and cathelicidin LL-37 at concentrations of 25 μg/mL induced epithelial cells death. Results reported by Kim et al. [26], showed an almost undetectable cytotoxicity of cathelicidins at concentrations below 10 μg/mL against four different cell lines. Jacobsen et al. [38], estimated that P-novispirin, a synthetic protegrin analog, exhibited reduced cytotoxicity at amounts lower than 100 μg/mL compared to protegrin -1, which was strongly cytotoxic even at the lowest concentrations.

The influence of ovine ANE on neutrophil secretory activity has not been previously evaluated, our study constitutes the first research on this topic. Other studies showed that an autologous rabbit extract of antimicrobial peptides decreased neutrophil activity [28], whereas a heterogenous one may cause an elevation of this activity [39]. We evaluated that ANE addition decreased both enzymatic and reactive oxygen species (ROS) response of isolated neutrophils both before and after surgical procedure. These results indicated that, similarly as in rabbit neutrophils, autologous antimicrobial extract acts anti-inflammatory.

According to Mansour et al. [40], AMP display a wide range of immunomodulatory activities, including pro- and anti-inflammatory responses, promoting wound healing and cellular differentiation. The polarization of macrophages to M1 phenotype appeared to be involved in the release of pro-inflammatory factors, development of a chronic inflammatory response and, eventually, a fibrotic response related to scar tissue formation. On the other hand, anti-inflammatory mediators released by M2 macrophages are present in tissue during the regeneration process. Thus, a phenotypic switch from M1 to M2 is considered a potential strategy for mitigating the effect of inflammation [4]. Our study revealed that under the influence of ANE, MDM polarized into anti-inflammatory macrophages, which was confirmed by functional changes, namely, a decreased superoxide and NO generation and increased arginase activity, coupled with morphological changes similar to the anti-inflammatory phenotype. As estimated previously, decreased generation of superoxide and nitric oxide together with increased arginase activity in MDM are essential for the healing process [41,42]. In our findings, we based the characterization of MDM phenotype on the functional analysis and morphological changes in comparison with cultures treated with LPS (as M1) or DEX (as M2c) [23]. According to Lock et al. [1], measuring the activation of immune cells provides information as to the functional outcome. High ratios of pro- to anti-inflammatory factors indicates that the material would likely induce a severe foreign body response, while high anti- to pro-inflammatory ratios predict potential positive healing outcomes in vivo. Pro-inflammatory effects may be characterized among others by the production of nitric oxide and ROS. Activation studies often rely on a comparison against factors that are known to activate immune cells to provide some clinical context i.e., LPS [1].

The last part of this experiment included stimulation of cultured MDM with an autologous neutrophil degranulation product (DGP). As estimated previously, extracellular neutrophil products are efficient regulators of many cellular functions and interactions between inflammatory cells. For example, neutrophil antimicrobial functions are not required during the implantation of a sterile implant and these cells play an immunomodulatory role rather than antimicrobial one. Therefore, the major role of activated neutrophils at inflammatory sites is to regulate and shape the inflammatory response. Neutrophil proteases may positively or negatively influence inflammatory reactions, among others, by modulating the activity of mediators released from necrotic cells. Thus, these neutrophil compounds may represent promising therapeutic targets for the treatment of conditions characterized by extensive neutrophil activity [43]. For this reason, we decided to evaluate the influence of DGP on MDM obtained from control sheep and sheep five months after implantation. Our study revealed that following treatment with DGP MDM adopted pro-inflammatory phenotype with increased NO and superoxide generation and decreased arginase activity in comparison with unstimulated MDM (BCS group) and DEX group. Moreover, MDM presented pro-inflammatory features in microscopic evaluation. According to Hussen et al. [21], bovine DGP polarized monocyte-derived macrophages towards a mixed macrophage phenotype with enhanced antimicrobial function. The influence of ovine DGP on macrophages differentiation has not been evaluated to date.

In the recent study Ng et al. [44] summarized previously conducted experiments with long-term use of Ti implants and concluded that the concentration of Ti in plasma and organs was related to both surface and time of implantation. In the study on rabbits, the serum and urine concentrations of Ti were evaluated for 12 months [45].

In another study on rabbit model, the Ti plate was removed after 1, 3, 6, 12, or 24 months after implantation and the obtained results revealed that Ti accumulation in the organs was highest at 3 months after implantation, and then gradually decreased. Ng et al. [44] conducted the experiment for 6 months as a time point of the expected maximum release of Ti particles from the implants due to friction with the implant drills during screw hole preparation or due to friction with the securing screws during placement. After this time the implant was expected to be stable; therefore, no further significant changes occurred after 6 months. In the study of Shelly et al. [46], on rats implanted bilaterally with a Ti graft in the femur, two experiments were conducted for the assessment of potential neurotoxicity of Ti particles: the first as a short-term study (5 weeks) for titanium biodistribution determination, and the second as a long-term study up to 10 months for measurement of Ti in brain and serum. Frisken et al. conducted the study of Ti release into the organs following the insertion of implants into the ovine mandibles and showed no significant release of Ti particles up to 3 months, which confirmed the successful integration of a single threaded screw type implant. On the basis of the literature and our previous findings, we decided to conduct the short-term study to evaluate the role of neutrophils in the process of implantation. Thus, the first time point T1 compared to control T0 was chosen for detection of changes in secretory response of circulating neutrophils as the main component of early-inflammatory response after surgery and implant insertion. Additionally, to assess the role of long-term contact of the host cells with implant, since many authors reported adverse effects during Ti implantation [8,12,13,14], we decided to evaluate this problem and in our experiment there were no adverse reactions up to 5 months after implantation of this biomaterial. This study provides knowledge on the implant–host immune interaction beyond the basic assessment of biocompatibility. The obtained results provided information on long-term interactions between Ti implants and host immune cells which could be translated into human medicine. However, assessment of the activity of circulating neutrophils and MDM at additional time points after implantation of a Ti plate will enrich knowledge about the interactions between the host and the implant.

## 5. Conclusions

This study revealed the lack of any undesirable effects either local, systemic, or involved in activity of some components of WBC up to 5 months after Ti implant insertion. This is a continuation of previous investigations, and these findings completed the knowledge about host–biomaterial interactions and potential mechanisms involved in these interaction.

Our findings provide knowledge about possible medical applications of neutrophil derived products.

Ovine antimicrobial neutrophil extract (ANE) did not exert any deleterious effect in contact with studied cells and may be a promising tool for the enhancement of the healing process through restriction of an undesirable inflammatory response.

The obtained results have shown that ANE can act as modulator of the secretory activity of neutrophils, and as anti-inflammatory stimulator of MDMs. These differences in cellular responses could be used in new autologous blood-derived products for regenerative medicine, since decreased generation of RONS with increased arginase activity are known to be essential for healing.

DGP caused macrophage polarization to pro-inflammatory phenotype for enhancement of host defense mechanisms.

## Figures and Tables

**Table 1:** Plasma concentration of haptoglobin (g/L) and fibrinogen (g/L) taken from sheep before implantation (**A**), immediately after surgery (**B**), and 7 days (**C**) after surgery.

Acute. Phase Protein	Before Surgery (A)	1h after Surgery (B)	7 Days after Surgery (C)
Haptoglobin (g/L)	0.20 ± 0.20	0.35 ± 0.33	0.22 ± 0.21
Fibrinogen (g/L)	4.10 ± 1.0	4.15 ± 0.81	4.44 ± 0.25

## Data Availability

The data presented in this study are available upon request from the corresponding author.
